# Investigating NAC Transcription Factor Role in Redox Homeostasis in *Solanum lycopersicum* L.: Bioinformatics, Physiological and Expression Analysis under Drought Stress

**DOI:** 10.3390/plants11212930

**Published:** 2022-10-31

**Authors:** Nagendra Rai, Krishna Kumar Rai, Manish Kumar Singh, Jagdish Singh, Prashant Kaushik

**Affiliations:** 1 Indian Institute of Vegetable Research (IIVR), Varanasi 221305, UP, India; 2 Department of Botany, Institute of Science, Banaras Hindu University, Varanasi 221005, UP, India; 3 Independent Researcher, 46022 Valencia, Spain

**Keywords:** drought stress, antioxidants, NAC, gene expression, bioinformatics

## Abstract

NAC transcription factors regulate stress-defence pathways and developmental processes in crop plants. However, their detailed functional characterization in tomatoes needs to be investigated comprehensively. In the present study, tomato hybrids subjected to 60 and 80 days of drought stress conditions showed a significant increase in membrane damage and reduced relative water, chlorophyll and proline content. However, hybrids viz., VRTH-16-3 and VRTH-17-68 showed superior growth under drought stress, as they were marked with low electrolytic leakage, enhanced relative water content, proline content and an enhanced activity of enzymatic antioxidants, along with the upregulation of NAC and other stress-defence pathway genes. Candidate gene(s) exhibiting maximum expression in all the hybrids under drought stress were subjected to detailed in silico characterization to provide significant insight into its structural and functional classification. The homology modelling and superimposition analysis of predicted tomato NAC protein showed that similar amino acid residues were involved in forming the conserved WKAT domain. DNA docking discovered that the SlNAC1 protein becomes activated and exerts a stress-defence response after the possible interaction of conserved DNA elements using Pro^72^, Asn^73^, Trp^81^, Lys^82^, Ala^83^, Thr^84^, Gly^85^, Thr^86^ and Asp^87^ residues. A protein–protein interaction analysis identified ten functional partners involved in the induction of stress-defence tolerance.

## 1. Introduction

In the 21st century, agriculture, crop growth and productivity are being redundantly affected by reiterative environmental pollution, prompting severe threats to global food security for ever-growing populations [1]. In Asia, where most farmers routinely practise rainfed agriculture, drought stress has become a daunting challenge evoking crop failure, yield losses and livestock death [2]. Plants activate many cellular, physiological, biochemical and molecular responses to confront drought stress-induced oxidative damage by regulating the expression of several stress-responsive genes, such as proline/glycine betaine, thus, improving photosynthesis and water-use efficiency [3]. The coordinated regulations of defence-related genes and the accumulation of osmolytes under drought stress are provoked by the expression of functional regulatory proteins that instigate an extensive reprogramming of transcriptome and metabolome. These functional regulatory proteins, i.e., transcription factors (TFs), play a central role in regulating the complex gene regulatory network of signal transduction and stress-defence pathways [4].

No Apical Meristem (NAM), Arabidopsis Transcription Activation Factor (ATAF), and Cup-Shaped Cotyledon (CUC) transcription factors, collectively called NAC TFs, are one of the most prominent families of TFs, which play a significant role in the transcriptional modulation and reprogramming of plant stress response [5]. Researchers have well reported that the expression of NAC TFs is well induced upon several abiotic as well as biotic stresses, and recent molecular studies using transgenic overexpressed, knockout/knockdown lines have well demonstrated that apart from NAC TFs, members of AP2/ERF, WRKY, MYB and bZIP TFs are widely involved in the modulation of plant developmental processes under drought stress [6].

The members of NAC TF proteins contain N-terminal DNA-binding NAC domain with five sub-domains, designated as A-E domains, of which domains A, C and D are remarkably conserved compared to B and E and highly variable C-terminal transcriptional activation domain rich in serine, threonine, proline and glutamine amino acid residues [7]. Extensive investigations in Arabidopsis, rice, and other plants have confirmed the role of NAC TFs under stress conditions [5]. Whole genome expression profiling and RNASeq analysis have led to the identification of putative NAC genes that are upregulated under biotic and abiotic stress conditions. The overexpression of rice NAC1, NAC2, NAC4 and NAC45 and Arabidopsis NAC019, NAC055 and NAC072 has increased plant defence response against multiple abiotic stresses [7]. Similarly, TaNAC2a overexpressing transgenic tobacco lines exhibited improved shoot/root biomass compared to wild lines under drought stress conditions [8]. Furthermore, two wheat NAC genes, i.e., GRAB 1 and GRAB 2, have enhanced the growth and productivity of wheat plants by controlling the expression of the wheat dwarf virus [9].

Tomato (*Solanum lycopersicum* L.) is a multipurpose vegetable crop widely cultivated around the globe. At the same time, it is highly susceptible to various abiotic/biotic stresses, of which drought stress significantly impacts its growth and productivity [10]. Therefore, deciphering the molecular determinants of drought stress tolerance is essential for improving agricultural production, although several NAC TFs have been reported in tomatoes that are upregulated under stress conditions [11]. However, little investigation has been made regarding the functional characterization of NAC TFs in tomatoes compared to rice and Arabidopsis [12]. Therefore, the present work mainly focuses on providing comprehensive knowledge about NAC genes’ structural and functional attributes in tomato hybrids. Here, we analyse and validate the role of three putative SlNAC1 genes and some stress-responsive genes in the modulation of the stress-defence response of tomato hybrids subjected to different drought stress treatments.

Furthermore, the function of these genes was also validated in tolerant and susceptible genotypes, which were used as “positive checks”. They were also used as a parent in the breeding program to develop these hybrids. The candidate gene(s) exhibiting maximum expression in all the hybrids under drought stress were then subjected to detailed in silico characterization in terms of the conserved residues involved in DNA binding, signalling cascades, DNA–protein interaction, their chromosomal location and other overlapping functions to provide new insight into the molecular function, structural and biological process of candidate NAC gene associated with drought tolerance response in tomato [13]. Eventually, this information can be utilized in other solanaceous plants for the identification/development of functional genes/markers for the genetic improvement of important agronomic traits through molecular breeding or transgenic approaches, in order to accelerate crop growth and yield under drought stress conditions.

## 2. Results

### 2.1. Evolutionary Analysis of NAC Protein

**Figure 1:**
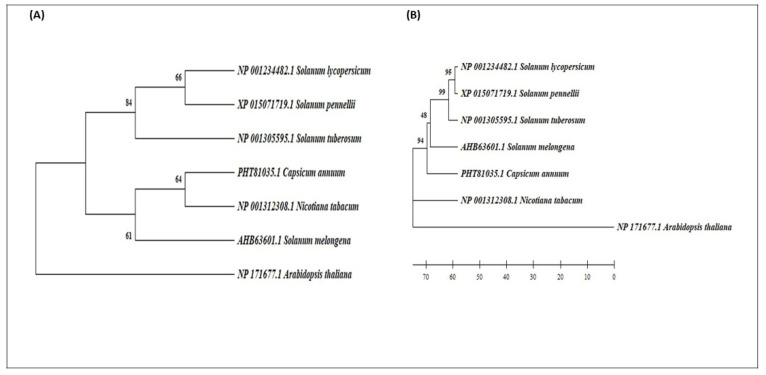
Phylogenetic tree showing (**A**) neighbour-joining (**B**) maximum parsimony of *Solanum lycopersicum* SlNAC1 protein among different members of the solanaceous family and model plant *A. thaliana*.

The phylogenetic analysis among solanaceous NAC protein family and model plant *A. thaliana* was performed against the non-redundant (nr) database. An unrooted tree was built by aligning the sequences showing maximum identity and query cover percentage, using MEGA 7.0 employing standard methods. The tree topologies generated by both algorithms revealed the monophyletic origin of SlNAC1 with *Solanum pennelli* and its close resemblance with *Solanum tuberosum* (Figure 1A,B).

**Figure 2:**
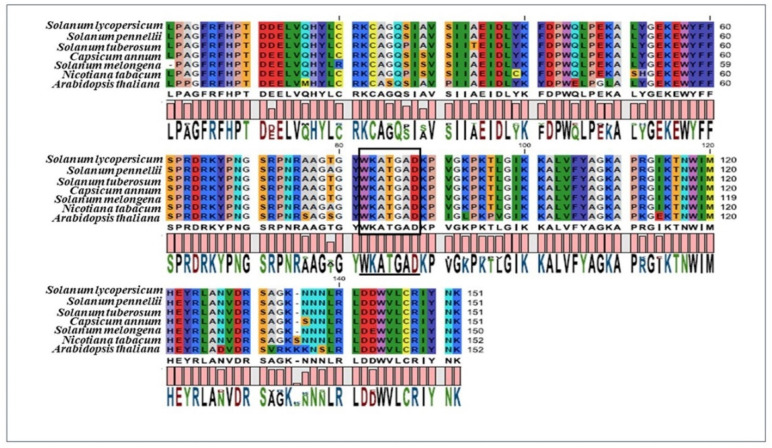
Multiple sequence alignment of conserved functional NAC domain region among different members of the solanaceous family and model plant *Arabidopsis thaliana* in the present study. Multiple sequence alignment analysis confirmed that the slightest discrepancies occurred in the WKAT motif during evolution and might exert a functional role in the interaction of NAC proteins with *cis*-regulatory elements.

Further, SlNAC1 was distinguished from other NAC members by forming an individual clade with a total bootstrap value of 94%, thus, indicating the recent expansion of SlNAC1 from other members of the solanaceous family. Inspection of the functional domain of SlNAC1 protein using a multiple sequence alignment analysis revealed maximum conservation of core amino acid residues around the WKATGAD region (Figure 2).

### 2.2. Gene Structure and Conserved Motifs Analysis

**Figure 3:**
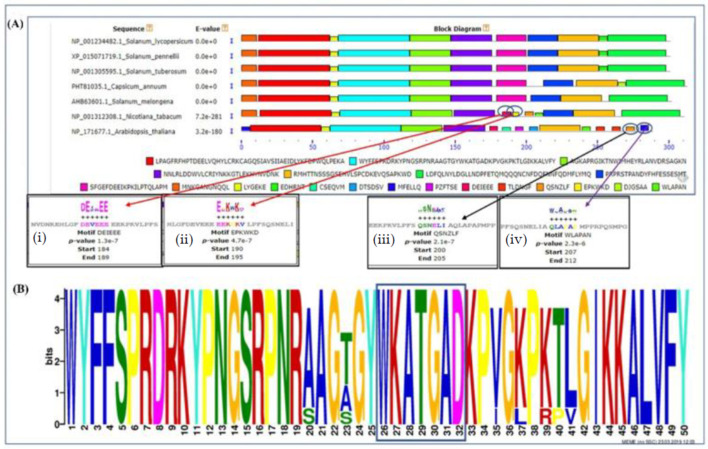
(**A**) The conserved motif analysis shows the distribution of common and uncommon motifs found in *S. lycopersicum*, *S. pennelli*, *S. tuberosum*, *C. annum*, *S. melongena*, *N. tabacum* and *A. thaliana*. (**B**) The sequential logo of motif 1.

The conserved motifs of NAC proteins in tomatoes were analysed by scanning the SlNAC1 proteins for the presence of putative motifs through the MEME analysis tool. Twenty-one motifs showed vital conservation around the WKAT motif across all the members of the NAC protein family. The result also illustrated that the phylogenetically related members had similar motif distribution patterns, thus, demonstrating structural and functional conservation among NAC proteins (Figure 3A).

The reproducibility and reliability of the identified motifs were confirmed using the *E*-value that corresponds to the frequency of the occurrences of the motif and the *p*-value which calculates the likelihood of their existence, and in the present study, the N-terminal of SlNAC1 protein (Figure 3A) was denoted by motif 1 (WYFFSPRDRKYPNGSRPNRAAGTGYWKATGADKPVGKPKTLGIKKALVFY; *p*-value 2.1e-64) The multiple sequence alignment results of NAC proteins corroborate the motif analysis result substantiating the conservation of core residues, as reflected in our motif distribution analysis. Interestingly, the presence of some specific motifs was also observed in other NAC protein family members, for instance, in *N tabacum* motif 2 (Figure 3A(i)) (DEVEEE; *p*-value 1.3e-7) and motif 3 (Figure 3A(ii)) (EEKPKV; *p*-value 4.7e-7), whereas in *A thaliana* motif 4 (Figure 3A(iii)) (QSNELI; *p*-value 2.1e-7) and motif 5 (Figure 3A(iv)) (QLAPAP; *p*-value 2.3e-6) they were observed at the C-terminal end (Figure 3A). These additional motifs within the same group may be due to sequence divergence during the evolutionary processes. The logo diagram of motif 1 representing the WKAT motif showing maximum conservation among all the solanaceous NAC members is depicted in Figure 3B.

### 2.3. Prediction of Conserved Functional Domains

Imposition of SlNAC1 protein to ExPASy PROSITE and InterProScan online tool (http://prosite.expasy.org) (assessed on 15 July 2022) revealed the presence of available signature sequence at the N-terminal end constituting the NAC domain region, thereby confirming its belonging to the NAC gene superfamily. Additionally, an analysis of SlNAC1 protein through InterProScan also clearly indicated that the putative SlNAC1 protein belongs to the NAC superfamily containing DNA-binding domain ranging from 1 to 301 amino acid residues. The results of the PROSITE and InterProScan analysis were also corroborated by the multiple sequence analysis performed for core signature sequences revealing vital conservation across all the members (Supplementary Materials, Figure S1).

### 2.4. Modelling and Superimposition of the NAC Domain

**Figure 4:**
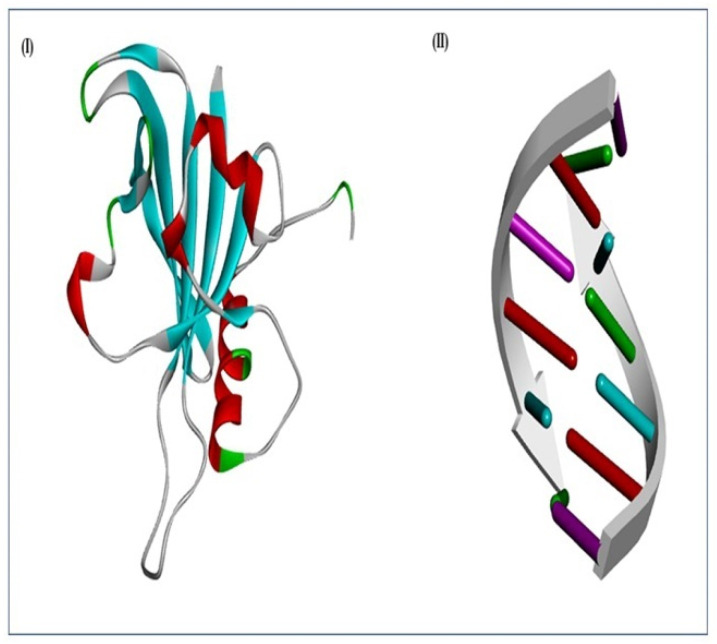
Homology modelling of (**I**) SlNAC1 and (**II**) DNA motif (5′-CATGTG-3′).

In the present study, a total of five models (SlNAC1) were generated and the model (Figure 4) exhibiting the lowest RMSD (Cα trace) and DOPE score compared to the respective templates was used for the interaction study (Table 1). The modelled SlNAC1 protein displaying the best score values was submitted to PMDB (PM0082340) (Supplementary Materials, Figure S2). Often, proteins of divergent evolution and distinct sequences exhibit structural resemblance; therefore, to test this hypothesis, the predicted SlNAC1 protein was superimposed on the respective templates using the SALIGN web server. The superimposition results indicated a high similarity of the modelled SlNAC1 protein with 1 UT7, 4 DUL and 3 ULX, as confirmed by their RMSD values viz., 1.74, 2.20, 2.94 A° alpha carbon and 1.73, 2.17, 2.92 A° around the backbone, indicating vital conservation of NAC domains across the divergent NAC superfamily (Supplementary Materials, Figure S3A(I–III)). The distinguishing topology for the modelled protein by the 3ULX template may be due to substitutions of some amino acid residues during evolution.

### 2.5. Statistical Validation of the SlNAC1 Model

The statistical validation of the predicted SlNAC1 model protein generated through the DS Modeller was performed in terms of probability density functions (PDFs) and discrete optimized protein energy (DOPE) scores. The geometry of the resultant SlNAC1 model was assessed using RAMPAGE and PROCHECK, which showed that 98% of the amino acid residues were observed in the most favoured regions (Table 2). Further, a PROCHECK analysis also predicted that 91.4% (expected 90%) of the residues occurred in the active region (Supplementary Materials, Figure S3B(I,II)). The ProSA analysis verified the superiority of the predicted SlNAC1 model over individual templates in terms of minimum Z score, thus, indicating that the expected model was very close to the template structures (Table 2; Supplementary Materials, Figure S3B(I,II)). In addition, the ERRAT analysis of the predicted model displayed a maximum score (60.17) compared to the respective templates, affirming superior atomic interactions in the resultant model (Table 2). The quality of SlNAC1 was also verified by a Resolution by Proxy (RESPROX) and Qualitative Model Energy Analysis (Q-MEAN), which significantly attested that the modelled protein had a better atomic resolution that allows more feasible long-range interaction in the expected model compared to the respective templates (Table 2). The quantitative validation of the predicted SlNAC1 model showed helical type structure 76 (50%), beta 58 (38%) and 18 (11%) helix type configuration with the observed H bond energy (−1.9, SD = 1.0) against expected (−2.0, SD = 0.8), which further attested the superiority of the predicted model over the templates.

### 2.6. DNA–Protein Docking

**Figure 5:**
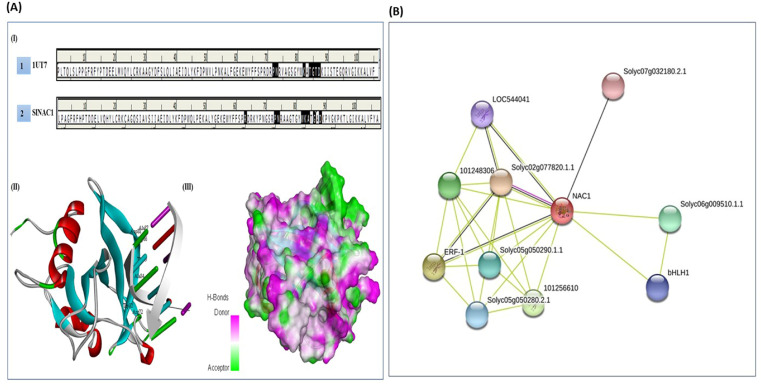
(**A**) (**I**) Docking analysis of SlNAC1 and 1UT7 with DNA motif. PNWKATGTD and RPNWKATGAD of SlNAC1 and 1UT7 interacted with DNA motif. (**II**) Docked SlNAC1 with DNA motif. (**III**) Three-dimensional surface view for docked complex in terms of hydrogen bonds donor and acceptor groups. (**B**) Protein–protein interaction network of SlNAC1 with other protein family members.

The molecular docking analysis was performed through the HexDoc server using the most reliable SlNAC1 model (Figure 5A(I)), sufficing all the crucial energy parameters with the 3D-modelled DNA element (Figure 5A(II)). The result indicated that the most stable and favourable docked complex has the binding energy (E_total_ = −1416.42 Kcal/mol; Supplementary Materials, Figure S4), which confirms that the NAC proteins are activated upon binding with the specific DNA elements through conserved WKAT motifs (Figure 5A(I)). The docking results further identified the key amino acid residues involved in the interaction as Arg^63^, Pro^73^, Asn^74^, Trp^82^, Lys^83^, Ala^84^, Thr^85^, Gly^86^, Ala^87^ and Asp^88^ (Figure 5A(I,II)), as compared to the template structure (1UT7), in which the interacting residues were Pro^71^, Asn^72^, Trp^81^, Lys^82^, Ala^83^, Thr^84^, Gly^85^, Ala^86^ and Asp^87^ (Figure 5A), thus, indicating the vital condensed nature of the core amino acid residues of WKAT motifs.

### 2.7. Protein–Protein Interaction

The functional protein–protein interaction was assessed using the STRING server at a medium confidence level from the values of the shell of interactors (Figure 5B). At a medium confidence score, SlNAC1 was found to have a strong interaction with Solyc04g009440.2.1 (score value 0.780) protein, which is an uncharacterized protein with a possible role in regulating NAC protein expression. The SlNAC1 protein also interacted with the ethylene-responsive transcription factor 1 protein, showing its significant role in regulating gene expression during fruit ripening and abiotic stresses. It is also involved in the modulation of stress signal transduction pathways with a score value of 0.777 from the first shell on interactors. Further, the SlNAC1 protein also showed interaction with the AP2 transcription factor family (101256610; score value 0.780), which is involved in the regulation of a wide array of plant physiological and molecular responses under various abiotic stresses, and the p450 protein involved in multiple biosynthetic and xenobiotic pathways under stress conditions (Solyc06g076160.2.1; score value 0.776). Apart from these, the SlNAC1 protein also interacted with three uncharacterized proteins viz., Solyc05g050280.2.1 (score value 0.701), Solyc05g050290.1.1 (score value 0.701) and Solyc06g009510.1.1 (score value 0.730) with glycoside hydrolase 3 (GH3) domain and are believed to be members of the phytohormone family of proteins contributing significantly in the induced biological activity of phytohormones under stress conditions (Figure 5B; Supplementary Materials, Table S1).

### 2.8. Gene Ontology Analysis and Subcellular Localization

To assess the biological, molecular function and subcellular localization, the predicted SlNAC1 protein was analysed by the CATH and Gene3D server, which uses a clustering algorithm to identify gene ontology (GO) terms. The GO terms under biological function were regulation of transcription (GO: 0006355); regulation of hyperosmotic and salinity response (GO:0042538); response to salicylic acid and water deprivation (GO: 0009751; GO: 0009414); and proline biosynthesis (GO: 0006561) under stress condition (Supplementary Materials, Figure S5A(I)), whereas the GO terms associated with molecular functions (Supplementary Materials, Figure S5A(II)) were DNA binding (GO: 0003677) and transcription factor activity (GO: 0003700). The Cello2GO results predicted that the SlNAC1 protein is mainly localized in the nucleus (37.4%; score value 1.869) and mainly intricated in regulating DNA-binding and transcription factor activity, whereas 22.5% of SlNAC1 proteins are located in the cytoplasmic region (score value 1.124) involved in various biosynthetic and metabolic processes (Supplementary Materials, Figure S5B). On the other hand, a cellular component analysis revealed that the predicted proteins have a wide range of functions, as inferred by the results of protein–protein interaction study.

### 2.9. Membrane Integrity, Tomato Fruit Vigour and Productivity

**Figure 6:**
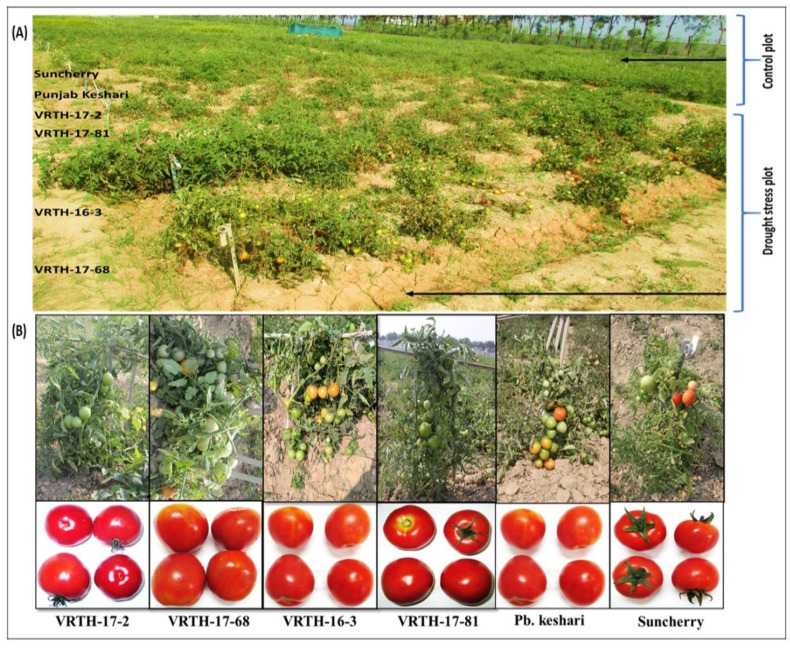
(**A**) Field screening of six genotypes, i.e., four hybrids and two parental lines, as positive controls for drought stress tolerance. All six genotypes were also grown under well water conditions, serving as a control plot. A typical irrigation pattern was followed for both plots until the plants reached their vegetative stage. At the onset of the vegetative stage, the irrigation of the stress plot was stopped. However, the control plot was irrigated regularly. (**B**) The relative performance of all four hybrids and positive controls under drought stress conditions.

Tomato hybrids, along with their respective parents grown under irrigation deficit conditions (Figure 6A), exhibited symbolic physiological changes compared to control irrigation (Table 3). Among the hybrids, VRTH-16-3 and VRTH 17-68 demonstrated enhanced physiological stability, as evident by their increased relative water content (15.2–20.1%; 16.9–19.1%) and decreased electrolytic leakage (53.8–61.01%; 56.5–70.0%) at 60 and 80 days of irrigation deficit condition compared to other hybrids (Figure 6B; Table 3). Similarly, in terms of length, width and total soluble solids (TSS), fruit vigour showed notable differences among all the hybrids/parents exposed to irrigation deficit conditions. However, VRTH-16-3 and VRTH 17-68 hybrids showed superior fruit length (45.5–50.5%; 35.1–42.6%), fruit width (48.4–48.9%; 37.8–40.8%) and TSS (27.5–38.6%; 26.9–34.3%) at 60 and 80 days of irrigation deficit condition compared with other hybrids and control (Table 3). Moreover, the productivity of hybrids in terms of the number of fruits per plant (NFPP), single fruit weight (SFW) and yield per plant (YPP) was also significantly reduced under irrigation deficit conditions compared to control irrigation. Nonetheless, maximum NFPP, SFW and YPP was also observed for the hybrids VRTH-16-3 (56.2–61.0%; 48.4–52.6%; 73.1–77.6%) and VRTH-17-68 (47.4–52.1%; 42.5–53.2%; 65.4–67.7%), respectively, at 60 and 80 days of irrigation deficit condition (Table 3).

### 2.10. Photosynthesis, Nutritional Analysis and Antioxidant Capacity

**Figure 7:**
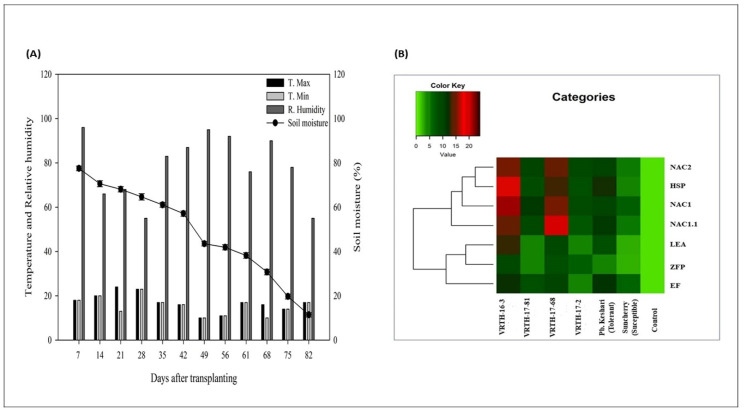
(**A**) Relative humidity (RH) (%), maximum temperature (T. max; °C), minimum temperature (T. min; °C) and soil moisture of experimental site during the investigation. (**B**) Relative expression of stress-responsive genes indicated in the form of heat map. Colour corresponds to the expression level of transcripts with low, intermediate and high expression.

The effect of irrigation deficit condition on photosynthetic pigment contents in the hybrids is consistent with the fruit vigour, productivity and membrane integrity results. As shown in Table 4, maximum chlorophyll and carotenoid contents were exhibited by VRTH-16-3 (20–37.6%; 30.7–54.8%) and VRTH-17-68 (20–22.7%; 25.6–37.5%) at 60 and 80 days of irrigation deficit condition (Table 4; Figure 7A). Correspondingly, the nutritional analysis of tomato hybrids revealed substantial differences in lycopene and ascorbic acid content under irrigation deficit conditions. The nutritional content was significantly increased under irrigation deficit conditions, where a maximum increase in lycopene and ascorbic acid content was observed for VRTH-16-3 (34.5–54.1%; 37.6–44.7%) and VRTH-17-68 (22.8–46.2%; 15.6–27.5%) subsequently at 60 and 80 days of irrigation deficit condition (Table 4). Furthermore, the antioxidant capacity of the hybrids/parents showed a similar pattern, as observed in the case of the nutritional analysis under both irrigation deficit and control irrigation conditions. The osmolyte proline and enzyme catalase was increased in all the hybrids, where maximum increment was observed for VRTH-16-3 (30.5–53.5%; 35.6–72.7%) and VRTH-17-68 (34.9–52.2%; 29.6–59.5%) subsequently at 60 and 80 days of irrigation deficit condition (Table 4), together with minimum lipid peroxidation (40.2–56.8%; 26.8–49.2%) and hydrogen peroxide (45–73%; 38.7–64.2%) generation subsequently at 60 and 80 days of irrigation deficit condition (Table 4).

### 2.11. Expression Analysis of Defence-Related Genes

To gain functional insight into the mechanism that induces drought stress tolerance in tomato hybrids, we examine the expression pattern of essential genes related to the stress-defence pathway. The genes viz., NAC 1, NAC1.1, NAC 2, heat shock protein (HSP), late embryogenesis abundant protein (LEA), zinc finger protein (ZFP) and elongation factor protein (EF) were selected for their possible role in the regulation of transcription, protein folding and activation of signal transduction pathways under various abiotic stresses (Figure 7B). The qRT analysis of the above genes was performed among all the tomato hybrids/parents at 80 days of water deficit condition. In the present study, the relative abundance of transcript encoding NAC1 (45.2–66.6%), NAC1.1 (39.9–54.6%) and NAC 2 (48.8–74.3%) was significantly increased more prominently in VRTH-16-3 and VRTH 17-68 compared to VRTH-17-2 and VRTH-17-81, as well as respective parents at 80 days of irrigation deficit condition. Similarly, the fold change of HSP (38.7–51.4%) and ZFP (22.4–30.9%) was increased in VRTH-16-3 and VRTH 17-68 and down-regulated in VRTH-17-2 and VRTH-17-81, as well as respective parents at 80 days of irrigation deficit condition (Figure 7B). Furthermore, the expression of ZFP and EF also showed a differential response in all the hybrids, where maximum upregulation was observed in VRTH-16-3 and VRTH 17-68. However, both LEA (20.3–28.9%) and EF (18.4–25.4%) were also significantly induced in VRTH-17-2 and VRTH-17-81 hybrids, as well as in tolerant parent Punjab Kesari. Altogether, the result indicated that the expression of the critical genes of the signal transduction pathway was more prominent in VRTH-16-3 and VRTH 17-68 hybrids compared to VRTH-17-2 and VRTH-17-81, which could be the possible reason behind the improved physiological growth, increased productivity and enhanced antioxidant capacity of VRTH 16-3 and VRTH 17-68 hybrids, thus, labelling them as drought-tolerant hybrids compared to their respective counterparts.

## 3. Discussion

In the present study, we tried to characterize SlNAC1 TFs using in silico approaches functionally. Then, we validated their role through a gene expression analysis in regulating physiological, biochemical and molecular drought stress responses in four tomato hybrids, along with respective drought-tolerant/susceptible parents. Initially, the presence of the NAC domain in the SlNAC1 protein was envisaged and established by a PROSITE and InterProScan analysis. The target sequence was then subjected to evolutionary computation, which revealed a close phylogenetic relationship among orthologs, monophyletic origin with *S. pennelli* and close resemblance with *S. tuberosum* NAC proteins. However, other members of the tomato family, such as *S. melongena*, *C. annum* and *N. tabacum*, form a separate cluster.

Interestingly, when SlNAC1 was compared with *A. thaliana*, the phylogenetic tree showed three distinct clusters showing *S. lycopersicum*, *S. pennelli* and *S. tuberosum* in one cluster and *S. melongena*, *C. annum* and *N. tabacum* in another cluster, whereas *A. thaliana* was out-clustered from the group (Figure 1A,B). The high resemblance among the NAC genes of tomato and potato might be due to their common ancestral origin, thus, sharing 97.1–100% homology and identity [13,14]. The results were also corroborated by multiple sequence alignment (Figure 2) done in the present study, which further confirms that the core residues around the WKAT motif in *S. lycopersicum*, *S. pennelli* and *S. tuberosum* are highly conserved compared to those of *S. melongena*, *C. annum and N. tabacum*, thus, indicating that the slightest disturbance occurred during the evolution [15,16].

The DNA–protein interaction ability of any transcription factors relies on the presence of functionally conserved motifs, specifically at the N-terminal domain, that are displayed as core residues capable of regulating various signalling pathways other than DNA–protein interaction [17]. Twenty-one motifs were predicted using MEME suit (Figure 3A), of which five motifs can be classified as A-E type, as they marked the presence of a complete DNA-binding domain and C-terminal activation domain [18]. In contrast, the remaining 16 motifs do not contain an entire DNA-binding domain; however, they exhibit location matching with the above five motifs [19]. We also identified two conserved motifs in all the members of the NAC family. First, the WKATGAD motif (Figure 3B) at the N-terminal domain is biochemically necessary for DNA–protein interaction and nuclear shuttling [20]. Furthermore, it has been reported that WKAT motifs can also modulate protein–protein interaction, thus, improving plant growth and development against pathogen attack and abiotic stresses [21]. Second, the LVFY motif (Figure 3B) at the C-terminal domain is essential for the activation/expression of transcription and protein–protein interaction [20].

The motif and phylogenetic analysis revealed that the identified genes are functional homologs of the SlNAC1 protein, thereby confirming the close evolutionary origin of the identified proteins. Several researchers have reported that genes with diverse, active roles might evolve from the same evolutionary process [22,23,24,25]. In recent years, evidence has suggested that structurally different homologs of NAC TFs play a crucial role in improving plant defence response when expressed in a tissue-specific manner [5].

The present study predicted the 3D structure of the SlNAC1 protein by choosing template proteins with the maximum query coverage and sequence identity using BLASTP search against the RCSB protein data bank (http://www.rcsb.org/) (assessed on 15 July 2022) [26]. Three template proteins viz., Template 1—PDB ID: 1UT7 (sequence identity 70%; query coverage 52%); Template 2—PDB ID: 3ULX (sequence identity 74.5%; query coverage 56%); and Template 3—PDB ID: 4DUL (sequence identity 70%; query coverage 52%) were used for modelling SlNAC1 protein. A large body of literature has indicated that the protein model generated using homology modelling usually suffers from poor bond angles, inappropriate bond lengths and unfavourable angle contacts [27]. A good protein model should have minimum energy to maintain appropriate bond angle geometry and, thus, normalize the close angle contacts [27]. In the present study, out of five models generated, the model (Figure 4) with a low DOPE score (−14,933.81; Table 1) was selected for interaction studies, as a model with such a score has minimum steric collision and strain. Therefore, they are much more stable than models with high DOPE scores [28]. The superimposition of modelled proteins with template proteins is also an alternate way to predict their relative accuracy and sequence homology [29]. Consequently, the superpose program was used for the 3D alignment of the predicted SlNAC1 model with the respective templates. The predicted model showed high structural (Figure 4) and sequence (Figure 4) similarity, as indicated by the root mean square deviation (RMSD) values of Cα superimposition (1.74, 2.20 and 2.97), suggesting that the predicted 3D structure of SlNAC1 protein is similar to the templates (Supplementary Materials, Figure S3A) [30].

The SlNAC1 model was then subjected to a quantitative analysis to verify/validate its topology and geometry. The qualitative estimation was performed using RAMPAGE. The PDBsum server indicated that about 98% of the residue lies in the favoured region and 2% in the outlier region from the RAMPAGE server (Table 2). In contrast, from the PDBsum server, 91.4% were in the favoured area, and no residues were present in the disallowed region (Supplementary Materials, Figure S3B(I,II)). Generally, a model is considered reliable if 90% of the residues fall in the favoured area [31]; hence, this putative model can be exploited for future studies in tomatoes. The position of the core residues in the predicted model was determined using a Z-score energy plot analysis of the ProSA webserver (Supplementary Materials, Figure S3B(I,II)). The Z-score analysis results further confirmed the expected model’s stability and reliability by measuring the total energy deviated from the total points distributed from random confirmations [32].

To qualify as a satisfactory model, the predicted models should have an ERRAT score of at least 50 and a minimum Q-mean score. In contrast, the ERRAT score of the expected model was high (60.1) compared to the respective templates and the Q-mean score was −1.87, indicating a minimum error in the structure (Table 2). The ResProx program was also used to predict the atomic resolution of the forecast model, and the expected model exhibited a value of 1.98 compared to the respective templates (Table 2). Usually, the nuclear resolution is sorted out based on the correlation coefficient values between the observed and calculated atomic coordinates of the nuclear model, and a model with a minimum ResProx score (<2.0 Å) is considered a good model [33]. The analysis mentioned above evidently verifies and validates the reliability of the modelled SlNAC1 protein.

In the present study, the DNA–protein docking analysis of the predicted SlNAC1 protein with the DNA element (CACATG) through the HexDoc server indicated that the amino acid residues viz., Arg^63^, Pro^73^, Asn^74^, Trp^82^, Lys^83^, Ala^84^, Thr^85^, Gly^86^, Ala^87^ and Asp^88^ were exclusively involved in the interaction with the DNA element (Figure 5A(I,II)). Further, this server reveals the presence of three-way binding sites in the predicted SlNAC1 protein, of which the last binding site was found to be more prominent (Figure 5A). To validate the effectiveness of the DNA protein interaction of the predicted model, the template protein (1UT7) was docked with the DNA element as a reference [34]. The results of the DNA–protein interaction indicated close resemblance in the interacting residues of both modelled SlNAC1 and the reference template (1UT7), thus, confirming that the predicted SlNAC1 protein (Figure 5A(II)) was modelled with the right folds and domains [35,36]. Therefore, the expected SlNAC1 model structure was effectively submitted to the PMDB database with PMDB ID PM0082340.

The string analysis reveals ten functional partners that showed a direct interaction with the predicted SlNAC1 protein, where the highest interaction was observed with uncharacterized protein LOC544041 with the identity of 84.1%, which is believed to be a member of the AP2 transcription factor family involved in the regulation of growth and development in plants exposed to climate extremes [37]. Further, the predicted SlNAC1 protein also interacted with ERF1 (Solyc03g093610.1) protein with an interaction score of 0.777, which is involved in the modulation of the expression of several defence-responsive genes, transcriptional activator and the regulation of diseases resistance pathway [38]. Other functional protein partners observed in the analysis scored between 0.551 and 0.699 with unknown functions (Supplementary Materials, Table S1), which could be involved in several other metabolic pathways, as suggested by their KEGG annotation. The present study’s interaction results pinpointed the differential regulatory pattern of the SlNAC1 protein, which could be responsible for imparting resistance in plants under drought and other abiotic stress conditions. Therefore, the functional characterization of SlNAC1 protein and its interaction partners can further open a new realm for gaining valuable insights into this protein’s molecular and regulatory function in other crop plants.

The gene ontology (GO) analysis was also used to categorize the SlNAC1 protein functionally based on its cellular, biological and molecular function [39]. Following the previous reports [40], the GO terms under biological processes (Supplementary Materials, Figure S5A(I,II)) were the genes that were involved in salicylic acid signalling, proline biosynthesis and salinity stress response, which may have been upregulated after interacting with NAC proteins and henceforth might have resulted in the enhanced tolerance of tomato hybrids under drought stress condition [33]. The GO terms governing molecular function (Supplementary Materials, Figure S5B) were the genes involved in DNA binding and transcription activation that stimulated the expression of NAC and associated proteins, resulting in the increased tolerance of tomato hybrids under drought stress [33]. Furthermore, we also assessed the relative subcellular localization of SlNAC1 protein through the Cello2GO web server, which indicated that 37.4% of the protein is of nuclear origin and mainly involved in DNA binding and transcriptional activation activity, and 22.5% of the protein to cytoplasm imparting biosynthetic function, as suggested by the results of the gene ontology analysis.

Drought stress instigates severe physiological and biochemical changes in plants, which differentially vary in stress intensity, agronomic factors and plant developmental stages [41]. In this study, we observed the differential tolerance response of tomato hybrids to drought stress. Higher relative water content and lower electrolytic leakage were observed in VRTH-16-3 and VRTH-17-68 hybrids compared to their corresponding counterparts/respective parents, demonstrating the competency of these hybrids to withhold water loss (Table 3 and Table 4). Leaf RWC and EL are considered reliable indicators of the physiological strength of plants exposed to abiotic stresses. Higher RWC/lower EL values are regarded as an indication of enhanced stress tolerance. The plants exhibiting higher RWC and lower EL are more capable of reducing stress-induced oxidative damage than their counterparts [42]. Based on this notion, the tomato hybrids VRTH-16-3 and VRTH-17-68 were considered tolerant hybrids under drought stress. Compared to their corresponding counterparts, both of these hybrids also showed balanced assimilation/metabolic processes, as evident by their improved reproductive growth measured in terms of fruit length, fruit width, single fruit weight, the total number of fruits, total soluble solids and total yield (Table 3 and Table 4; Figure 6A,B).

The significant increase (Table 5) in the yield and related attributes of VRTH-16-3 and VRTH-17-68 hybrids may have been due to their improved ability to maintain efficient transcriptional control of stress-responsive genes’ increase in photosynthesis by avoiding transpiration [43]. Furthermore, increased catalase activity may have resulted in the enhanced scavenging of reactive oxygen species, particularly hydrogen peroxide, which might lead to decreased membrane damage in the tomato fruits. Correspondingly, low membrane damage and enhanced proline content could have significantly improved the fruit diameter, size and yield of the tomato hybrids VRTH-16-3 and VRTH-17-68 more efficiently than their respective counterparts and control.

Changes in plant metabolites are often associated with impairment in the photosynthetic process driven by accelerated water loss, thus, severely affecting the biosynthesis of essential nutritional components [44]. Our results have shown that in VRTH-16-3 and VRTH-17-68 hybrids, a positive correlation was observed between the RWC and biosynthesis of critical dietary components such as lycopene and ascorbic acid photosynthetic pigment contents, i.e., chlorophyll and carotenoids (Table 4). In contrast, the rest of the hybrids/parents showed a significant decrease in the nutritional components and chlorophyll and carotenoid contents. The enhanced biosynthesis of dietary components and photosynthetic pigment contents in the above hybrids could be due to their increased water-use efficiency, which improved their CO_2_ assimilation, thereby maintaining their photosynthesis and water loss under drought stress conditions [45]. Suppressing nutritional factors such as lycopene, ascorbic acid and other related traits under drought stress is a natural phenomenon that varies greatly depending on the plant’s idiotype and organ [42]. In this study, lycopene and ascorbic acid were affected more than total soluble solids (TSS), where the effect was observed less for the tomato hybrids VRTH-16-3 and VRTH-17-68. The firm level of lycopene and ascorbic acid content for these tomato hybrids could be due to the enhanced expression of stress-responsive genes that have strengthened the catalase activity, leading to improved drought stress tolerance [44]. 

A higher proline and lower malondialdehyde accumulation are considered the best biochemical markers for plant drought tolerance [45]. In our research, higher concentrations of proline and lower concentrations of malondialdehyde were observed in VRTH-16-3 and VRTH-17-68 hybrids compared to the rest of the plants (Table 4). Higher proline accumulation under stress conditions has improved stress tolerance in several plant species [46]. Drought-exposed plants suffer from an extensive loss of reducing power due to limited CO_2_ assimilation that results in the accumulation of reactive oxygen species; thus, maintaining a higher level of antioxidant enzymes becomes imperative to prevent oxidative damage. We observed a higher activity of catalase enzyme coupled with a low level of H_2_O_2_ for VRTH-16-3 and VRTH-17-68 hybrids compared to their corresponding counterparts (Table 3 and Table 4). The increased catalase enzyme activity in the above hybrids could have been due to improved physiological activity, such as low electrolytic leakage, decreased lipid peroxidation and enhanced chlorophyll content, and the up-accumulation of proline has rendered the harmful effect of generated H_2_O_2_ [46].

The overexpression of specific stress-responsive genes has been implicated in improving drought stress tolerance by increasing plants’ antioxidant capacity, thus, enhancing the ROS scavenging and reducing the toxic effect of drought stress on plants [47]. We observed that all three variants of NAC proteins viz., NAC1, NAC1.1, NAC2 and HSP showed high constitutive expression in VRTH-16-3 and VRTH-17-68 hybrids compared to their respective counterparts and were clustered in one group. In contrast, LEA, ZFP and EF genes showed moderate expression (Figure 7B). The enhanced tolerance of VRTH-16-3 and VRTH-17-68 hybrids could be due to the stimulatory growth role of the NAC genes in drought tolerance that might have enhanced the expression of several defence-responsive genes that have rendered better ion/osmotic homeostasis, induced antioxidant capacity and reduced water loss, thereby minimizing drought stress-induced oxidative damages [48]. However, future studies are required to decipher the detailed functional mechanism of NAC genes that can further our understanding of their molecular roles in drought tolerance in tomato plants.

## 4. Materials and Method

### 4.1. Database Search, Sequence Retrieval and Comparative Phylogeny

The NCBI database was used to retrieve the sequence of tomato NAC1 protein (Locus: NP_001234482.1), and the NCBI BLAST server http://blast.ncbi.nlm.nih.gov/Blast.cgi (assessed on 15 July 2022) [49] was exploited to identify relevant sequential and functional homologues available for this protein in tomatoes. The NAC1 protein sequence showing maximum percentage identity and query cover was used for its functional characterization and phylogenetic analysis. The Bio-Edit tool [50] was used to analyse the sequence alignment and construct a phylogenetic tree using the MEGA 7 suite http://www.megasoftware.net (assessed on 15 July 2022) [51]. A multiple sequence alignment analysis of the selected protein was performed using a CLC bio workbench. In addition, functional NAC domain regions occupied by NAC proteins were analysed using InterProScan http://www.ebi.ac.uk/Tools/pfa/iprscan (assessed on 15 July 2022) [52], NCBI CDD server http://www.ncbi.nlm.nih.gov/Structure/cdd/cdd.shtml (assessed on 15 July 2022) [53] and ExPASy-Prosite scan http://prosite.expasy.org/scanprosite (assessed on 15 July 2022) [54] analysis.

### 4.2. Identification of Conserved Motifs

MEME (Multiple EM for Motif Elicitation) https://meme-suite.org/meme/ (assessed on 18 July 2022) Suite 5.0.5 was exploited to analyse the functional motifs in NAC1 protein. A MEME analysis was performed by default selection parameters [55]. The MAST alignment tool was further used for the structural annotation of conserved motifs to identify functional motif patterns from other orthologs.

### 4.3. Homology Modelling of Tomato NAC Protein and Superimposition

The homology modelling of the SlNAC1 protein was carried out by the MODELLER software package (version 7.7) [56]. The template proteins viz., the structure of the conserved domain of Arabidopsis NAC (PDB ID: 1UT7), the crystal-structural of the conserved domain of rice stress-responsive NAC1 (PDB ID: 3ULX) and Arabidopsis NAC019 NAC domain crystal (PDB ID: 4DUL) were used to generate the 3D structure of proteins using the MODELLER module of Discovery Studio 3.0 [56]. The query SlNAC1 protein was refined for Cα traces using the ModRefiner tool http://zhanglab.ccmb.med.umich.edu/ModRefiner (assessed on 18 July 2022) [57]. The best model structure was then used for superimposition and docking study [58].

### 4.4. Qualitative Analysis of Modelled SlNAC1 Protein

The qualitative evaluation of SlNAC1 proteins and templates was analysed through the PROCHECK module of the PDBSum server http://www.ebi.ac.uk/pdbsum/ (assessed on 15 August 2022) [59] and further validated by the RAMPAGE server http://mordred.bioc.cam.ac.ik/~rapper/rampage.php (assessed on 15 August 2022) [60]. ProSA https://prosa.services.came.sbg.ac.at/ [61], Q-mean https://swissmodel.expasy.org/qmean/ (assessed on 15 August 2022) [62], RESPROX (Resolution by Proxy) and ERRAT http://services.mbi.ucla.edu/ERRAT/stats/ (assessed on 15 August 2022) [63] were also exploited to confirm the reliability of the modelled SlNAC1. A VADAR (Volume, Area, Dihedral Angle Reporter) http://vadar.wishartlab.com/ analysis was used for a quantitative estimation of the modelled protein (assessed on 15 August 2022) [64]. The DALI http://ekhidna2.biocenter.helsinki.fi/dali/ ProTSAV meta server http://www.scfbio-iitd.res.in/software/proteomics/protsav.jsp (assessed on 15 August 2022) [65] and Verify3D web server http://servicesn.mbi.ucla.edu/Verify3D/ were used to confirm and validate the stability of our modelled SlNAC1 protein (assessed on 15 August 2022). The best-modelled SlNAC1 protein was further submitted to an online repository of PMDB [66].

### 4.5. DNA–Protein Docking Analysis

The Hex 8.0 molecular docking server was used to analyse DNA–protein interaction [67]. The DNA sequence to structure server http://www.scfbio-iitd.res.in/software/drugdesign/bdna.jsp was used to model DNA sequences that specifically interact with the modelled SlNAC1 protein (assessed on 19 August 2022). The docking was performed using default parameters, and the docked complex was analysed using Discovery Studio 3.0 to reveal critical amino acid residues involved in DNA–protein interaction.

### 4.6. Protein Interactive Network, Functional Annotation and Subcellular Localization

The STRING server (Search Tool for the Retrieval of Interacting Genes/Proteins) database version 10.0 http://string-db.org/ (assessed on 10 September 2022) [68] was used to identify the interacting partners of SlNAC1 protein using default parameters. The CATH/Gene3D server http://www.cathdb.info/ (assessed on 10 September 2022) [69] and FunFHMMER http://www.cathdb.info/search/by-funfhmmer (assessed on 10 September 2022) [70] were used for the gene ontological (GO) analysis. The REVIGO http://revigo.irb.hr/ (assessed on 10.09.2022) [71] and CELLO2GO web servers http://cello.life.nctu.edu.tw/cello2go/ (assessed on 10 September 2022) [72] were used for finding their subcellular localization.

### 4.7. Active Site Prediction

The amino acid residues involved in creating the active site for mediating DNA–protein interaction were analysed using the metapocket server http://metapocket.eml.org (assessed on 13 September 2022) [73].

### 4.8. Plant Materials and Stress Condition

The tomato plants were subjected to drought stress at the experimental farm of the Indian Institute of Vegetable Research, Varanasi, UP, India, during the post-rainy season (October to March). The physical and chemical properties of soil and weather during the experimental period are represented in Table 5, respectively. During the experiment, the mean day and night temperature (DNT) and mean relative humidity (RH) were 22/32 °C and 75/70% with a 12 h light/dark period. The mean water content in the soil during the entire experimental period was 40–45% of field capacity. The tomato hybrids viz., VRTH 17-2, VRTH 17-68, VRTH 17-81 and VRTH 16-3 and their respective parents viz., Punjab Keshari (drought tolerant, DT) and Suncherry (drought susceptible, DS) were screened for their ability to tolerate drought stress under open field condition. The randomized block design (RBD) was used to design experiments with three treatments and replicated thrice. Each treatment (two treatments (T1 and T2) and control) had six plots, and each plot had 35 plants. The control plots were irrigated as per the requirement of the agronomic condition, whereas in the treatment plots (a) treatment 1 (T1), drought stress was imposed at a vegetative state in which irrigation was withheld for 60 days; (b) treatment 2 (T2), wherein the irrigation was withheld to the crops for 80 days (between 40 and 120 days after transplanting). The first and last rows were used as border rows in each treatment plot, including the control plot. In contrast, four central rows were used for recording the yield data and morphophysiological and biochemical evaluations at the end of the stress imposition. After completion of the experiment, the fully expanded third leaf from the top was stored at −80 °C for physiological, biochemical and molecular analysis. The fruit samples were analysed 14 days after the completion of 80 days of drought stress conditions when the tomato fruits attained the red stage.

### 4.9. Estimation of Leaf Water Potential and Chlorophyll Contents

The relative water content (RWC) was estimated following the standard protocol [74]. Leaf discs of a uniform size devoid of midrib were used to measure the fresh weight (FW), turgid weight (TW) and dry weight (DW) and the leaf RWC was calculated as per the standard equation. Chlorophyll contents were analysed by grinding fresh leaf samples in 80% acetone per the protocol [75]. The chlorophyll content was measured per Arnon’s equation [76] and expressed as mg g^−1^ FW. Total suspended solids (TSS) in tomato fruits were measured using a refractometer. Fruit diameter, single fruit weight, the number of fruits per plant and yield per plant were counted manually. All the measurements were carried out in triplicate.

### 4.10. Estimation of Electrolytic Leakage and Lipid Peroxidation

The electrolytic conductivity of leaf latchets was measured following the standard method devised by Deshmukh et al. [77]. The electrolytic leakage was recorded at 40 °C (C1) and 100 °C (C2) using a conductivity meter (Century Instruments, Chandigarh, India). The measurement of lipid peroxidation was performed using the standard method [78]. Fresh leaf samples were extracted in 10% tri-chloroacetic acid (TCA) containing 0.25% of 2-thiobarbituric acid, and the malondialdehyde level was calculated using the extinction coefficient of 155 mM^−1^ cm^−1^ and expressed as μmol g^−1^ FW.

### 4.11. Estimations of Lycopene and Ascorbic Acid

The lycopene content was estimated as per the standard protocol [79]. The tomato fruits were extracted in petroleum ether and acetone (3:2) simultaneously. After centrifugation, the OD of the supernatant was measured at 503 nm and expressed as mg of lycopene/100 g following the standard equation. The ascorbic acid [80] in the tomato fruit samples was estimated by titrating the fruit samples homogenized in 4% oxalic acid solution against 2, 6-dichlorophenol indophenol dye solution. The end point of the titration was measured with the development of a light pink colour, which persisted for 10 s. The ascorbic acid in tomato fruits was expressed as mg/100 g following the standard equation.

### 4.12. Measurements of Hydrogen Peroxide and Catalase Activity

The hydrogen peroxide content in the fresh samples was measured using the standard method [81]. The leaves were extracted in sodium phosphate buffer (pH 7.2) and 0.1% titanium sulphate, and the absorbance was measured at 410 nm. The hydrogen peroxide generated was measured using the extinction coefficient of 0.28 μM^−1^ cm^−1^ and expressed as μmol g^−1^ FW. As Bates et al. [82] described, the proline level in the leaf samples was estimated. Leaf samples were extracted in 3% sulfosalicylic acid and incubated at 100 °C for 1 h. After incubation, the samples were cool, and toluene was added. The absorbance containing acid ninhydrin was recorded at 520 nm and expressed as μg g^−1^ FW. The activity of the antioxidative enzyme catalase was assayed per the standard method [83]. The leaf samples were homogenized in 50 mM of Tris NaOH buffer (pH 8.0), and the absorption of the supernatant was recorded at 240 nm.

### 4.13. RNA Isolation and cDNA Preparation

TRIZOL reagent (Invitrogen, Waltham, MA, United States) was used to isolate total RNA following the manufacturer’s recommendations. The quality of the total RNA was checked using a nanophotometer (Implen, Westlake Village, CA, USA), and integrity was confirmed on 1.0% agarose gel electrophoresis. The isolated RNA (1.0 μg) was then used to synthesize the first strand of cDNA using an iScriptTM cDNA synthesis kit (Bio-Rad Laboratories, Hercules, CA, USA).

### 4.14. Real-Time (RT-PCR) Gene Expression Analysis

An SsoFast^TM^ EvaGreen^®^ Supermix detection chemistry (Bio-Rad) iQ5thermocycler (BioRad Laboratories, Hercules, CA, USA) was used for the real-time PCR analysis using gene-specific primers (Supplementary Materials, Table S2). The reactions were formulated in 20 μL, and relative quantification was carried out per the standard 2^−∆∆CT^ method given by Livak and Shmittgen [84], using *ACTIN* mRNA level as an internal control. The gene expression analysis was performed as three biological and technical replicates to generate a heat map.

### 4.15. Statistical Analysis

The statistical analysis was performed using two-way analyses of variance (two-way ANOVA) with two fixed factors, i.e., genotype and treatment. All the data were recorded in three biological replicates. The Duncan Multiple Range Test (DMRT) was used to compare means at a 0.05 significance level using SPSS 21.0 software, IBM Corporation, Armonk, NY, USA.

## 5. Conclusions

In the present study, the hybrids viz., VRTH-16-3 and VRTH-17-68 ameliorated drought-induced oxidative stress more efficiently by competently modulating their water-holding capacity, membrane damage, proline biosynthesis and up-accumulation of NAC genes, as well as other stress-responsive genes, compared to different hybrids. The present study confirmed that drought stress differentially modulated the expression of various stress-responsive genes, enzymes and metabolites in tomato hybrids. Likewise, the differential expression of NAC genes in the present study attested to their regulatory roles in ameliorating drought stress-induced oxidative damage in tomato hybrids by stimulating their physiological, biochemical and molecular defence response, thereby contriving the destructive effect of drought-induced oxidative stress. However, the molecular mechanisms behind this seminal role of NAC genes in tomatoes need to be addressed in detail.

Further, the pilot study’s bioinformatic analysis of SlNAC1 genes also provided valuable insight into their structural and functional classification and evolutionary relatedness with other members of NAC gene families. The docking analysis recognized that Arg^63^, Pro^73^, Asn^74^, Trp^82^, Lys^83^, Ala^84^, Thr^85^, Gly^86^, Ala^87^ and Asp^88^ residues improve NAC protein plant growth and developmental process under abiotic stress conditions. Furthermore, the generated SlNAC1 model was reliable in quantitative and qualitative assessment and can be exploited for future studies in other crop plants. Overall, the information generated in the present study will unravel the complex regulatory networks associated with NAC TFs in plants exposed to drought stress conditions. In addition, integrating NAC genes in the tomato breeding program will help breeders develop climate-smart crops with enhanced adaptability and survival under adverse conditions, thus, ensuring agricultural sustainability and global food security.

## Figures and Tables

**Table 1:** Molecular PDF energy, GA341 score and DOPE score for modelled SlNAC1.

Model Name	Molecular PDF Energy	GA341 Score	DOPE Score
Predicted model scores for NAC1			
MODEL 1 (B0001)	882.3424	1.00000	−14,933.8125
MODEL 2 (B0002)	874.4256	1.00000	−15,016.9257
MODEL 3 (B0003)	869.1204	1.00000	−15,057.3261
MODEL 4 (B0004)	841.9102	1.00000	−15,006.6411
MODEL 5 (B0005)	1043.384	1.00000	−14,987.5429

**Table 2:** Assessment of qualitative and quantitative scores for the modelled NAC1 protein and its comparison with the experimentally deduced (X-ray diffracted/NMR) structures.

S. No.	Protein Name	Q-Mean Score	Z Score	Overall Quality ERRAT Score	ReSProx	Rampage Results		
						Most Favoured (%)	Additionally, Allowed (%)	Outlier Region (%)
1.	SlNAC1 *S. lycopersicum* (NCBI ID: NP_001234689.1) (predicted model)	−1.87	−5.95	60.17	1.98	98.0	0.0	2.0
2.	Structure of the conserved domain of ANAC, a member of the NAC family of transcription factors (PDB ID: 1UT7) (Template 1)	0.76	−3.72	56.66	2.04	98.2	1.8	0.0
3.	Crystal structure of the conserved domain of rice stress-responsive NAC1 (PDB ID: 3ULX) (Template 2)	−3.16	−4.74	57.62	2.78	85.0	9.3	5.7
4.	ANAC019 NAC domain crystal form IV (PDB ID: 4DUL) (Template 3)	−2.90	−3.34	53.84	3.03	91.1	6.2	2.7

A good quality protein has ReSProx values ranging between 0 and 1.5, ERRAT score of more than 90, minimum Z and Q-mean scores occupying more than 98% of the residues in the most favoured region, less than 2% residues in additionally allowed region and 0% residues in outlier region in RAMPAGE analysis.

**Table 3:** Changes in physiological and yield-related attributes in tomato hybrids/parents under drought stress.

Hybrids/Parents	Treatments	RWC (%)	EL (%)	TSS (° Brix)	FL (cm)	Fw (cm)	SFW (g)	NFPP	Y/P (kg/Plant)
VRTH-17-2	Control	81.2 ± 0.48 ^c^	20.1 ± 0.40 ^c^	3.85 ± 0.25 ^bc^	4.30 ± 0.20 ^c^	4.63 ± 0.27 ^cd^	49.3 ± 1.01 ^d^	33.6 *±* 0.68 ^d^	1.70 ± 0.19 ^d^
	60 days	68.2 ± 0.43 ^b^	29.2 ± 0.45 ^c^	4.47 ± 0.22 ^b^	3.74 ± 0.23 ^cd^	4.29 ± 0.23 ^c^	35.0 ± 0.90 ^e^	22.3 ± 0.69 ^de^	0.81 ± 0.18 ^e^
	80 days	62.2 ± 0.46 ^b^	32.9 ± 0.29 ^c^	4.57 ± 0.29 ^b^	4.09 ± 0.19 ^c^	3.85 ± 0.19 ^d^	26.3 ± 0.78 ^f^	18.5 ± 0.30 ^e^	0.58 ± 0.13 ^e^
VRTH-17-68	Control	85.9 ± 0.38 ^b^	18.2 ± 0.41 ^d^	4.36 ± 0.28 ^b^	5.47 ± 0.27 ^ab^	5.74 ± 0.28 ^b^	64.4 ± 0.82 ^b^	54.7 ± 0.84 ^b^	3.55 ± 0.16 ^b^
	60 days	77.3 ± 0.44 ^a^	21.4 ± 0.46 ^e^	5.32 ± 0.25 ^a^	5.12 ± 0.22 ^b^	5.18 ± 0.15 ^b^	58.7 ± 0.35 ^c^	46.6 ± 0.85 ^b^	2.51 ± 0.19 ^b^
	80 days	70.3 ± 0.43 ^a^	28.3 ± 0.47 ^d^	5.57 ± 0.22 ^a^	5.23 ± 0.26 ^b^	5.07 ± 0.19 ^b^	51.3 ± 0.89 ^a^	35.2 ± 0.82 ^bc^	1.68 ± 0.15 ^d^
VRTH-17-81	Control	76.6 ± 0.43 ^d^	23.8 ± 0.49 ^b^	3.39 ± 0.20 ^d^	4.79 ± 0.20 ^c^	5.13 ± 0.28 ^c^	45.6 ± 0.76 ^d^	40.4 ± 1.00 ^bc^	1.74 ± 0.24 ^d^
	60 days	66.1 ± 0.58 ^c^	36.4 ± 0.42 ^a^	4.18 ± 0.27 ^b^	3.92 ± 0.22 ^cd^	4.74 ± 0.20 ^bc^	35.6 ± 0.89 ^d^	34.6 ± 0.91 ^c^	0.82 ± 0.22 ^e^
	80 days	59.0 ± 0.52 ^c^	40.6 ± 0.49 ^b^	4.62 ± 0.24 ^b^	4.16 ± 0.24 ^c^	4.34 ± 0.18 ^c^	25.2 ± 0.78 ^f^	24.5 ± 0.85 ^cd^	0.59 ± 0.11 ^e^
VRTH-16-3	Control	92.8 ± 0.33 ^a^	14.5 ± 0.48 ^e^	4.14 ± 0.17 ^ab^	6.57 ± 0.30 ^a^	6.89 ± 0.22 ^a^	75.3 ± 0.82 ^a^	66.6 ± 0.90 ^a^	5.11 ± 0.22 ^a^
	60 days	78.0 ± 0.45 ^b^	22.6 ± 0.52 ^e^	5.69 ± 0.21 ^a^	6.10 ± 0.29 ^a^	6.25 ± 0.34 ^a^	65.4 ± 0.95 ^b^	57.2 ± 0.68 ^b^	3.62 ± 0.29 ^b^
	80 days	70.9 ± 0.53 ^c^	28.8 ± 0.54 ^d^	5.62 ± 0.25 ^a^	6.03 ± 0.19 ^a^	5.93 ± 0.21 ^ab^	50.7 ± 0.66 ^cd^	42.3 ± 0.75 ^c^	2.16 ± 0.08 ^c^
Punjab Keshari (DT)	Control	79.8 ± 0.52 ^c^	20.8 ± 0.38 ^c^	3.54 ± 0.17 ^cd^	4.43 ± 0.25 ^c^	4.60 ± 0.28 ^cd^	54.6 ± 0.70 ^c^	42.6 ± 0.62 ^c^	2.61 ± 0.25 ^c^
	60 days	76.3 ± 0.40 ^a^	25.7 ± 0.43 ^d^	3.49 ± 0.29 ^c^	3.64 ± 0.32 ^cd^	3.71 ± 0.23 ^d^	42.9 ± 0.59 ^d^	35.1 ± 0.92 ^d^	1.42 ± 0.13 ^d^
	80 days	68.6 ± 0.47 ^a^	31.9 ± 0.63 ^c^	4.07 ± 0.20 ^c^	3.53 ± 0.27 ^d^	3.43 ± 0.22 ^e^	34.7 ± 0.68 ^e^	26.5 ± 0.76 ^c^	0.84 ± 0.12 ^e^
Suncherry (DS)	Control	77.9 ± 0.41 ^d^	29.5 ± 0.41 ^a^	3.73 ± 0.24 ^bcd^	4.40 ± 0.29 ^c^	4.47 ± 0.24 ^d^	40.4 ± 0.45 ^d^	37.2 ± 0.77 ^d^	1.78 ± 0.22 ^d^
	60 days	67.1 ± 0.54 ^bc^	32.6 ± 0.51 ^b^	4.10 ± 0.21 ^b^	3.32 ± 0.21 ^d^	3.22 ± 0.17 ^e^	33.7 ± 0.60 ^e^	35.1 ± 0.92 ^cd^	0.81 ± 0.17 ^e^
	80 days	63.2 ± 0.47 ^b^	44.3 ± 0.45 ^a^	4.76 ± 0.20 ^b^	3.00 ± 0.16 ^e^	3.03 ± 0.13 ^f^	24.0 ± 0.68 ^f^	20.2 ± 0.48 ^de^	0.62 ± 0.22 ^e^
Two-Way ANOVA									
Genotype		***	***	***	***	***	***	***	***
Treatment		***	***	***	***	***	***	***	***
Genotype × treatment		***	***	*	*	NS	*	NS	***

The values of relative water content (RWC), electrolytic leakage (EL). TSS: total suspended solid, FL: fruit length, FW: fruit width, SFW: single fruit weight, NFPP: number of fruits per plant, Y/P: yield/plant (means of three replicates ± SE in the same column with different letters are significantly different at the 0.05 level, according to Duncan’s multiple range test (*p* < 0.05). The letters are assigned by comparing all the values from (i) control plants. Then, the letters were assigned to 60-days treated plants by comparing all the values from (ii) 60-days drought stress plants and, finally, the letters were assigned to 80-days treated plants by comparing all the values from (iii) 80-days drought stress plants. Data were analysed with two-way ANOVA. * *p* ˂ 0.05; *** *p* ˂ 0.001; NS: not significant. DT: drought tolerant, DS: drought susceptible.

**Table 4:** Changes in nutritional and antioxidant capacity in tomato hybrids/parents under drought stress.

Hybrids/Parents	Treatments	Lycopene (mg g^−100^ FW)	Ascorbic Acid (mg g^−100^ FW)	Chlorophyll (mg g^−1^ FW)	Carotenoid (mg g^−1^ FW)	LPO (μM g^−1^ FW)	Proline (µg g^−1^ FW)	H_2_O_2_ (µM g^−1^ FW)	Catalase (µmol H_2_O_2_ Reduced min^−1^ mg^−1^ Protein)
VRTH-17-2	Control	2.40 ± 0.13 ^c^	7.65 ± 0.30 ^f^	4.57 ± 0.25 ^ab^	1.99 ± 0.17 ^b^	1.03 ± 0.17 ^d^	20.3 ± 0.53 ^f^	1.27 ± 0.19 ^ef^	5.30 ± 0.22 ^f^
	60 days	2.98 ± 0.16 ^c^	10.4 ± 0.44 ^e^	3.80 ± 0.25 ^bc^	1.65 ± 0.15 ^b^	1.84 ± 0.15 ^c^	42.2 ± 0.45 ^e^	1.84 ± 0.12 ^e^	6.07 ± 0.20 ^e^
	80 days	3.43 ± 0.15 ^d^	11.7 ± 0.35 ^de^	3.50 ± 0.27 ^bc^	1.39 ± 0.18 ^c^	2.46 ± 0.25 ^b^	55.5 ± 0.42 ^d^	2.84 ± 0.09 ^d^	11.5 ± 0.42 ^d^
VRTH-17-68	Control	4.60 ± 0.23 ^c^	12.9 ± 0.33 ^d^	5.41 ± 0.19 ^a^	2.14 ± 0.19 ^a^	1.01 ± 0.16 ^d^	22.0 ± 0.37 ^f^	1.14 ± 0.19 ^ef^	10.2 ± 0.41 ^d^
	60 days	6.22 ± 0.35 ^a^	15.0 ± 0.38 ^b^	4.23 ± 0.28 ^b^	1.44 ± 0.18 ^bc^	1.37 ± 0.16 ^d^	60.5 ± 0.47 ^c^	1.36 ± 0.10 ^ef^	15.1 ± 0.38 ^c^
	80 days	6.38 ± 0.40 ^a^	16.1 ± 0.38 ^b^	3.65 ± 0.27 ^bc^	1.43 ± 0.21 ^bc^	1.75 ± 0.16 ^c^	77.1 ± 0.60 ^b^	1.82 ± 0.18 ^e^	22.3 ± 0.48 ^b^
VRTH-17-81	Control	3.29 ± 0.17 ^d^	10.2 ± 0.45 ^e^	3.49 ± 0.29 ^bc^	1.75 ± 0.24 ^b^	1.70 ± 0.26 ^c^	18.9 ± 0.34 ^f^	1.56 ± 0.14 ^e^	4.61 ± 0.24 ^f^
	60 days	3.89 ± 0.43 ^c^	12.9 ± 0.28 ^d^	3.29 ± 0.19 ^c^	1.43 ± 0.27 ^bc^	2.74 ± 0.20 ^b^	35.7 ± 0.56 ^ef^	4.10 ± 0.31 ^b^	7.64 ± 0.36 ^e^
	80 days	5.00 ± 0.25 ^c^	14.5 ± 0.46 ^c^	1.40 ± 0.25 ^e^	0.55 ± 0.16 ^d^	3.47 ± 0.19 ^a^	49.4 ± 0.41 ^e^	5.90 ± 0.33 ^a^	8.90 ± 0.30 ^d^
VRTH-16-3	Control	4.49 ± 0.20 ^c^	14.2 ± 0.39 ^c^	5.45 ± 0.29 ^a^	2.52 ± 0.25 ^a^	1.27 ± 0.19 ^d^	21.3 ± 0.46 ^f^	1.04 ± 0.19 ^ef^	13.1 ± 0.40 ^cd^
	60 days	6.88 ± 0.29 ^a^	18.1 ± 0.25 ^a^	5.13 ± 0.20 ^a^	1.99 ± 0.15 ^ab^	1.49 ± 0.17 ^d^	63.6 ± 0.66 ^c^	1.16 ± 0.14 ^ef^	20.4 ± 0.45 ^b^
	80 days	7.48 ± 0.29 ^a^	19.5 ± 0.38 ^a^	4.50 ± 0.28 ^ab^	1.64 ± 0.21 ^c^	1.71 ± 0.18 ^c^	83.9 ± 0.74 ^a^	1.84 ± 0.19 ^e^	32.8 ± 0.67 ^a^
Punjab Keshari (DT)	Control	3.31 ± 0.20 ^d^	12.2 ± 0.41 ^d^	5.10 ± 0.16 ^a^	1.70 ± 0.27 ^bc^	1.24 ± 0.14 ^d^	20.3 ± 0.43 ^f^	1.37 ± 0.12 ^ef^	9.10 ±0.22 ^d^
	60 days	5.06 ± 0.19 ^b^	14.1 ± 0.44 ^c^	4.30 ± 0.27 ^ab^	1.46 ± 0.22 ^bc^	1.61 ± 0.24 ^c^	50.1 ± 0.60 ^d^	1.53 ± 0.12 ^e^	15.3 ± 0.47 ^c^
	80 days	5.67 ± 0.26 ^b^	14.3 ± 0.42 ^c^	3.68 ± 0.30 ^bc^	1.37 ± 0.19 ^bc^	2.02 ± 0.15 ^b^	68.3 ± 0.48 ^c^	2.42 ± 0.27 ^d^	24.0 ± 0.27 ^b^
Suncherry (DS)	Control	3.33 ± 0.20 ^d^	9.64 ± 0.41 ^ef^	4.30 ± 0.27 ^ab^	1.45 ± 0.21 ^bc^	1.65 ± 0.16 ^c^	16.6 ± 0.42 ^d^	1.68 ± 0.13 ^e^	4.20 ± 0.24 ^f^
	60 days	4.54 ± 0.27 ^cc^	12.1 ± 0.39 ^d^	3.20 ± 0.23 ^c^	0.90 ± 0.16 ^d^	2.36 ± 0.25 ^b^	27.4 ± 0.49 ^f^	3.16 ± 0.23 ^c^	6.90 ± 0.39 ^ef^
	80 days	5.42 ± 0.27 ^b^	12.6 ± 0.41 ^d^	2.82 ± 0.27 ^cd^	0.81 ± 0.13 ^d^	3.45 ± 0.25 ^a^	39.0 ± 0.55 ^ef^	5.09 ± 0.33 ^a^	8.93 ± 0.32 ^d^
Two-Way ANOVA									
Genotype		***	***	***	***	***	***	***	***
treatment		***	***	***	***	***	***	***	***
Genotype × treatment		**	*	*	*	***	***	***	***

The values of lycopene, ascorbic acid, chlorophyll, carotenoid, lipid peroxidation (LPO), proline, hydrogen peroxide (H_2_O_2_) and catalase (means of three replicates ± SE) in the same column with different letters are significantly different from each other at the 0.05 level, according to Duncan’s multiple range test (*p* < 0.05). The letters are assigned by comparing all the values from (i) control plants. Then, the letters were assigned to 60-days treated plants by comparing all the values from (ii) 60-days drought stress plants and, finally, the letters were assigned to 80-days treated plants by comparing all the values from (iii) 80-days drought stress plants. Data were analysed with two-way ANOVA. * *p* ˂ 0.05; ** *p* ˂ 0.01; *** *p* ˂ 0.001; NS: not significant. DT: drought tolerant, DS: drought susceptible.

**Table 5:** Physical and chemical properties of soil at the experimental site.

Texture	Sandy Loam
Sand (%)	50–55
Silt (%)	30–35
Clay (%)	15–20
Total nitrogen content (%)	170.11–187.55
Organic carbon content (%)	0.40–0.50
Phosphorous content (%)	9.49–10.38
Electrical conductivity (dSm^−1^)	0.35–0.40
pH	6.0–7.0

## Data Availability

Not applicable.

## Supplementary Materials

The following supporting information can be downloaded at: https://www.mdpi.com/article/10.3390/plants11212930/s1, Figure S1. Prediction of the location of NAC domain in SlNAC1 protein using InterProScan scan analysis; Figure S2. Submission of our predicted model to PMDB and assigned PMDB ID (PM0081904) for our submitted structure; Figure S3. Superimposition results represented with their respective global and local RMSD-values showing the structural conservation of SlNAC1 domain. Superimposition results revealed that SlDREB1were found to be structurally closer to 1UT7 template compared to 4DUL, 3ULX templates as evident from their variations for RMSD values. Sequence alignment between SlDREB1 with the respective templates (1UT7, 4DUL, 3ULX) predicts the synonymous (blue) and non-synonymous substitutions (white) (B) Qualitative analysis of predicted model using PROCHECK and ProSA analysis. (I) The stereo chemical spatial arrangement of amino acid residues in predicted model (SlDREB1) were compared with experimentally resolved structures (1UT7, 4DUL, 3ULX) through PROCHECK server. Most favoured regions are coloured red while additionally, generously and disallowed regions are indicated with yellow, light yellow and white fields respectively. (II) Qualitative estimation by ProSA server, that measures structural errors in each amino acid residues; Figure S4. Binding energy of docked complex of SlNAC1 protein with DNA element. Figure S5. Gene ontology analysis using ReviGO web server. The functional and significant GO terms involved in (I) biological processes and (II) molecular function are shown on scattered plot diagram using hypergeometric test distribution of SlNAC1. (B) The subcellular localization and functional gene annotation of SlNAC1 using CELLO2GO web server. The significant terms are represented in terms of their percentage contribution; Table S1. Functional annotation, accession ID and interacting score values for both first and second shell of interactors that form mutual interactive associative network along with SlNAC. The higher value of score indicate the more frequent interaction exist between two associated proteins; Table S2. Primers used for quantification of mRNA levels by qRT-PCR.
