# Correction: Rai et al. Investigating NAC Transcription Factor Role in Redox Homeostasis in *Solanum lycopersicum* L.: Bioinformatics, Physiological and Expression Analysis Under Drought Stress. *Plants* 2022*, 11*, 2930

**DOI:** 10.3390/plants15132049

**Published:** 2026-07-02

**Authors:** Nagendra Rai, Krishna Kumar Rai, Manish Kumar Singh, Jagdish Singh, Prashant Kaushik

**Affiliations:** 1 Indian Institute of Vegetable Research (IIVR), Varanasi 221305, UP, India; nrai1964@gmail.com (N.R.); krishna.rai4@bhu.ac.in (K.K.R.); mksagri90@gmail.com (M.K.S.); jsingh4@sify.com (J.S.); 2 Department of Botany, Institute of Science, Banaras Hindu University, Varanasi 221005, UP, India; 3 Independent Researcher, 46022 Valencia, Spain

In the publication [1], there was an error regarding the affiliation for Prashant Kaushik. The updated affiliation should be: Independent Researcher, 46022 Valencia, Spain.

The authors state that the scientific conclusions are unaffected. This correction was approved by the Academic Editor. The original publication has also been updated.
