# Supplementary material for: Investigating NAC Transcription Factor Role in Redox Homeostasis in Solanum lycopersicum L.: Bioinformatics, Physiological and Expression Analysis under Drought Stress
Source: Plants (Basel). 2022 Oct 31;11(21):2930. doi: 10.3390/plants11212930 (PMC9654907; doi:10.3390/plants11212930)
Supplement: Supplementary file 1 [file plants-11-02930-s001.zip › plants-1948338-supplementary.pdf]

Authors:

Nagendra Rai[a], Krishna Kumar Rai[a,b], Manish Kumar Singh[a], Jagdish Singh[a] and Prashant Kaushik[c*]

Article Title:

Investigating NAC transcription factor role in redox homeostasis in *Solanum lycopersicum* L.: Bioinformatics, physiological and expression analysis under drought stress

Affiliation

[1] Indian Institute of Vegetable Research (ICAR-IIVR), Post Box-01, P.O.-Jakhini (Shahanshahpur), Varanasi-221305 (Uttar Pradesh), India

[2] Department of Botany, Institute of Science, Banaras Hindu University, Varanasi, 221005, U.P. India

[3] Instituto de Conservación y Mejora de la Agrodiversidad Valenciana, Universitat Politècnica de València, 46022 Valencia, Spain

Author for correspondence

* Email: prakau@doctor.upv.es;

Supporting information's

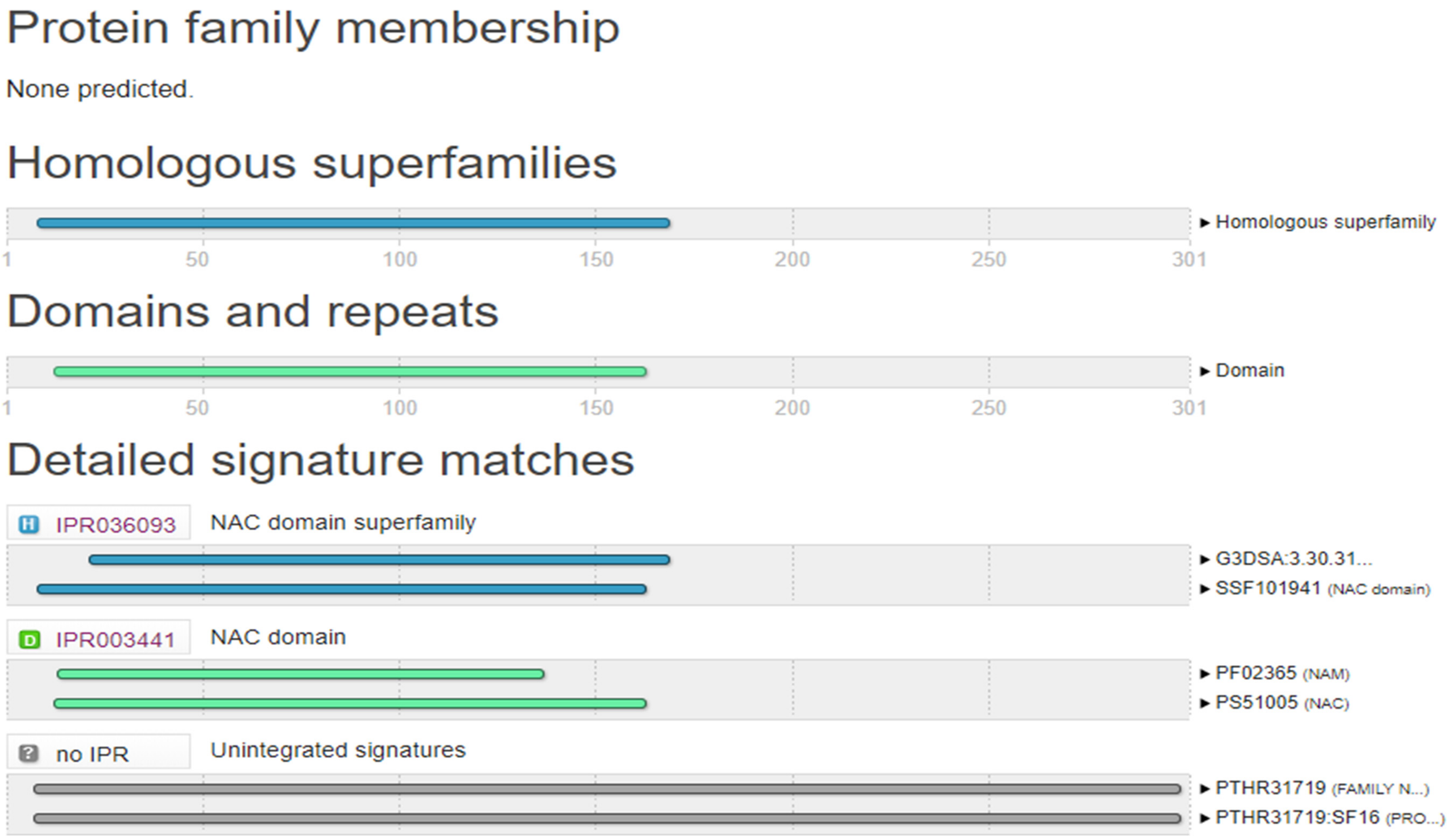

Supporting Information Figure S1 Prediction of the location of NAC domain in SlNAC1 protein using InterProScan scan analysis.

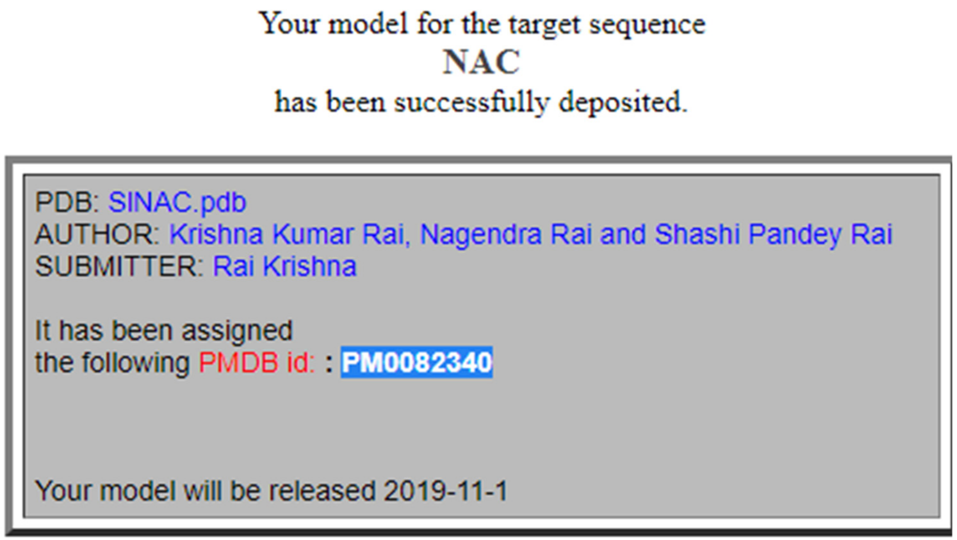

Supporting information Figure S2 Submission of our predicted model to PMDB and assigned PMDB ID (PM0081904) for our submitted structure

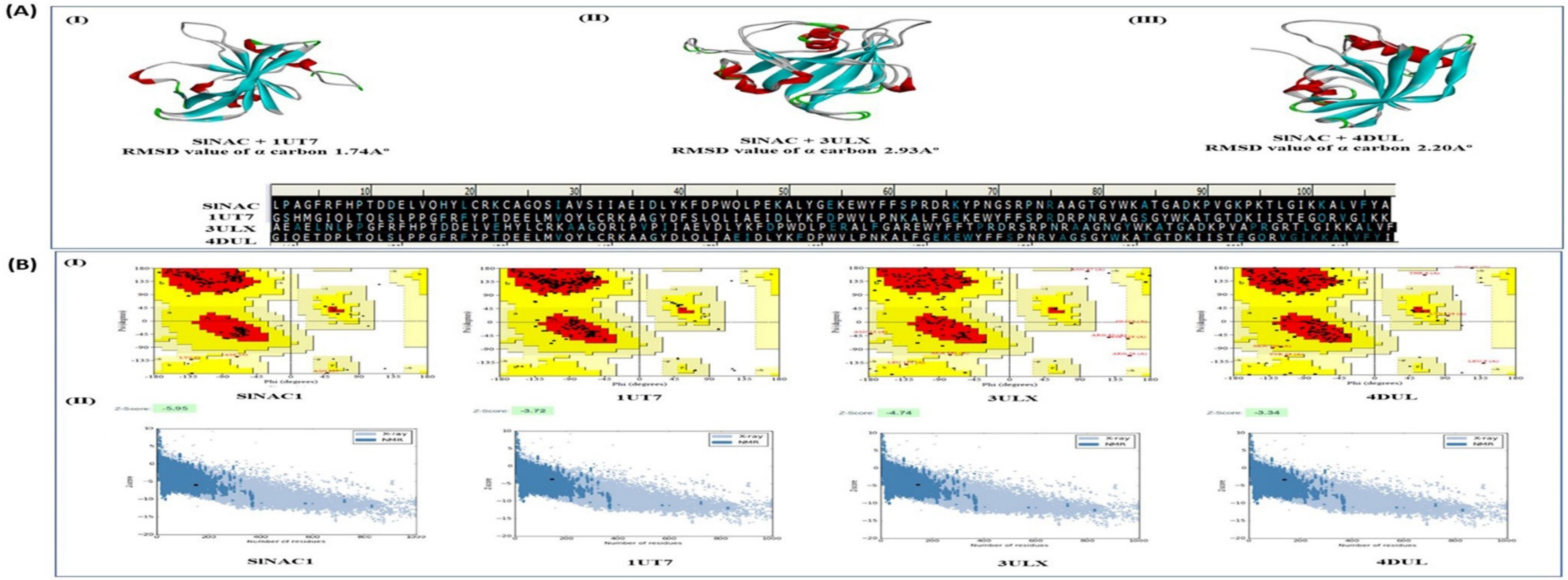

Supporting Information Figure S3. Superimposition results represented with their respective global and local RMSD-values showing the structural conservation of SlNAC1 domain. Superimposition results revealed that SlDREB1were found to be structurally closer to 1UT7 template compared to 4DUL, 3ULX templates as evident from their variations for RMSD values. Sequence alignment between SlDREB1 with the respective templates (1UT7, 4DUL, 3ULX) predicts the synonymous (blue) and non-synonymous substitutions (white) **(B)** Qualitative analysis of predicted model using PROCHECK and ProSA analysis. (I) The stereo chemical spatial arrangement of amino acid residues in predicted model (SlDREB1) were compared with experimentally resolved structures (1UT7, 4DUL, 3ULX) through PROCHECK server. Most favoured regions are coloured red while additionally, generously and disallowed regions are indicated with yellow, light yellow and white fields respectively. (II) Qualitative estimation by ProSA server, that measures structural errors in each amino acid residues

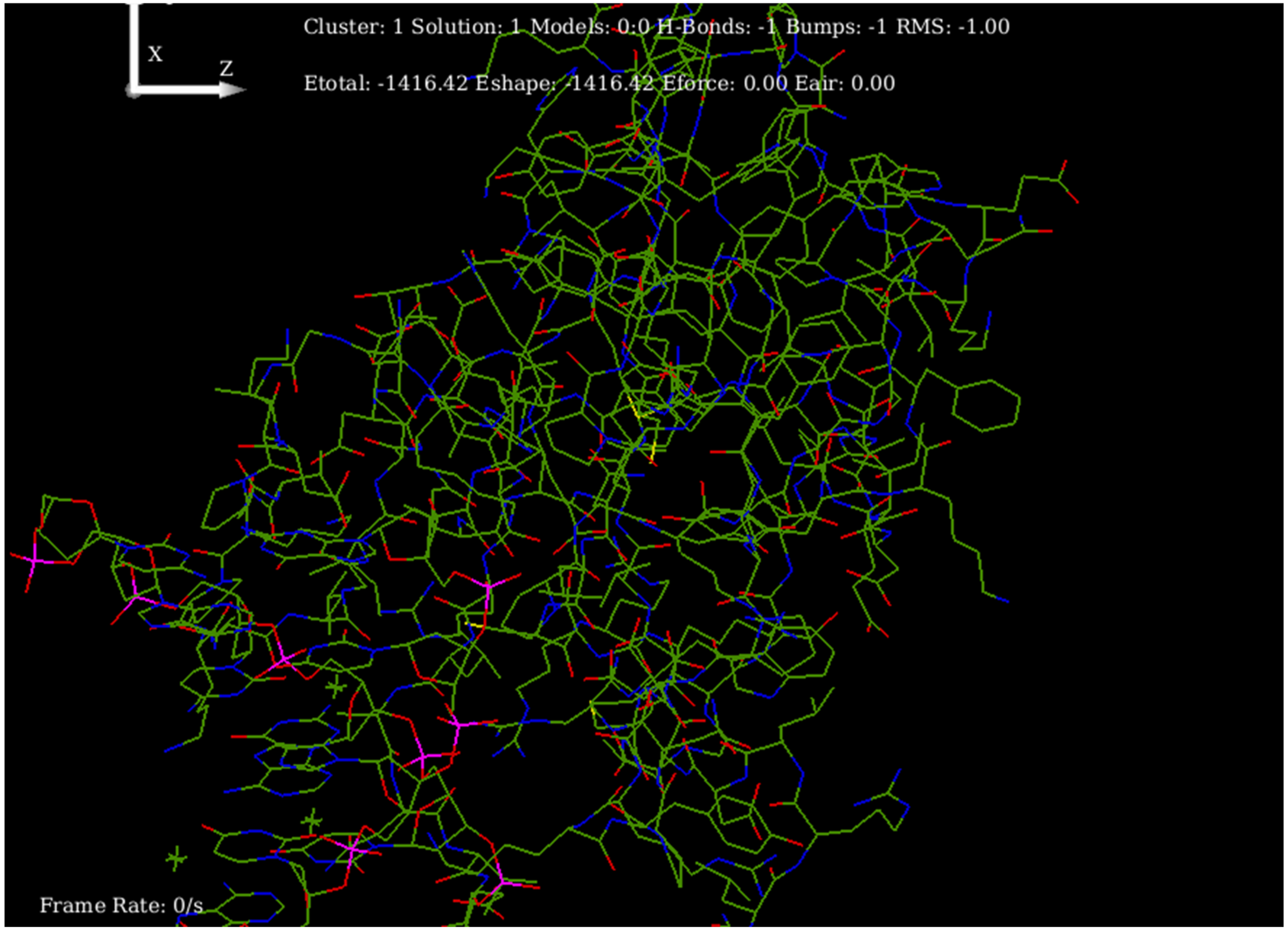

Supporting information Figure S4 Binding energy of docked complex of SlNAC1 protein with DNA element

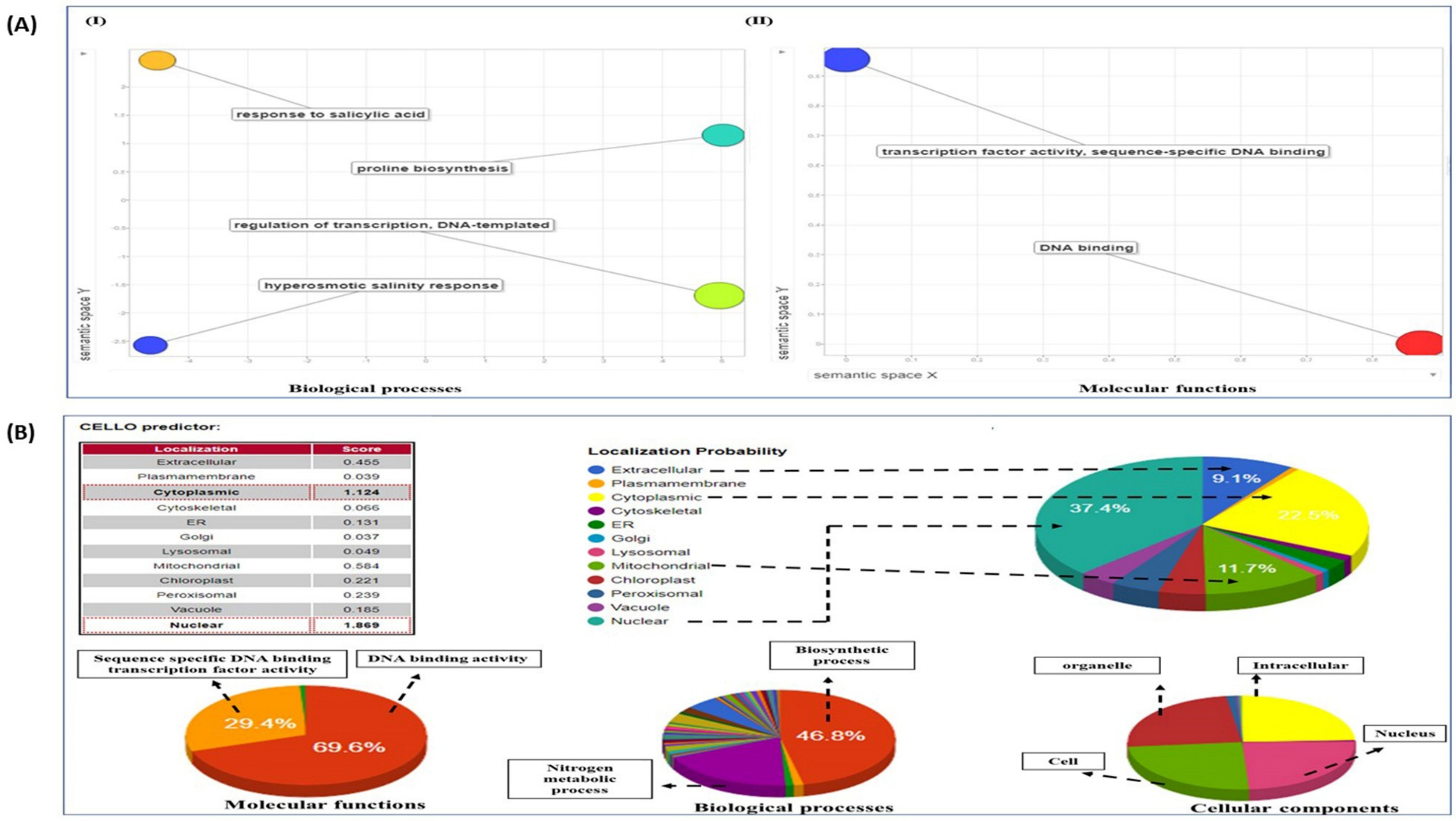

Supporting information Figure S5 Gene ontology analysis using ReviGO web server. The functional and significant GO terms involved in (I) biological processes and (II) molecular function are shown on scattered plot diagram using hypergeometric test distribution of SlNAC1. (B) The subcellular localization and functional gene annotation of SlNAC1 using CELLO2GO web server. The significant terms are represented in terms of their percentage contribution

Supporting Information Table S1 Functional annotation, accession ID and interacting score values for both first and second shell of interactors that form mutual interactive associative network along with SlNAC. The higher value of score indicate the more frequent interaction exist between two associated proteins.

Node1	Node2	Node1 accession	Node2 accession	Node1 annotation	Node2 annotation	Score
101248306	101256610	Solyc06g076160.2.1	Solyc08g007230.1.1	annotation not available	annotation not available	0.776
101248306	ERF-1	Solyc06g076160.2.1	Solyc03g093610.1.1	annotation not available	Ethylene-responsive transcription factor 1; Involved in the regulation of gene expression during fruit ripening, by stress factors and by components of stress signal transduction pathways. Transcription factor that binds to the GCC-box pathogenesis-related promoter element. Probably acts as a transcriptional activator and may be involved in disease resistance pathways (By similarity)	0.773
101248306	LOC544041	Solyc06g076160.2.1	Solyc05g052050.1.1	annotation not available	Uncharacterized protein; Pti4	0.583
101248306	NAC1	Solyc06g076160.2.1	Solyc04g009440.2.1	annotation not available	NAC domain protein; NAC domain protein 1	0.776
101248306	Solyc02g077820.1.1	Solyc06g076160.2.1	Solyc02g077820.1.1	annotation not available	annotation not available	0.776
101248306	Solyc05g050280.2.1	Solyc06g076160.2.1	Solyc05g050280.2.1	annotation not available	annotation not available	0.699
101248306	Solyc05g050290.1.1	Solyc06g076160.2.1	Solyc05g050290.1.1	annotation not available	annotation not available	0.699
101256610	101248306	Solyc08g007230.1.1	Solyc06g076160.2.1	annotation not available	annotation not available	0.776

101256610	NAC1	Solyc08g007230.1.1	Solyc04g009440.2.1	annotation not available	NAC domain protein; NAC domain protein 1	0.776
101256610	Solyc02g077820.1.1	Solyc08g007230.1.1	Solyc02g077820.1.1	annotation not available	annotation not available	0.78
101256610	Solyc05g050280.2.1	Solyc08g007230.1.1	Solyc05g050280.2.1	annotation not available	annotation not available	0.699
101256610	Solyc05g050290.1.1	Solyc08g007230.1.1	Solyc05g050290.1.1	annotation not available	annotation not available	0.699
ERF-1	101248306	Solyc03g093610.1.1	Solyc06g076160.2.1	Ethylene-responsive transcription factor 1; Involved in the regulation of gene expression during fruit ripening, by stress factors and by components of stress signal transduction pathways. Transcription factor that binds to the GCC-box pathogenesis-related promoter element. Probably acts as a transcriptional activator and may be involved in disease resistance pathways (By similarity)	annotation not available	0.773
ERF-1	NAC1	Solyc03g093610.1.1	Solyc04g009440.2.1	Ethylene-responsive transcription factor 1; Involved in the regulation of gene expression during fruit ripening, by stress factors and by components of stress signal transduction	NAC domain protein; NAC domain protein 1	0.777

				pathways. Transcription factor that binds to the GCC-box pathogenesis-related promoter element. Probably acts as a transcriptional activator and may be involved in disease resistance pathways (By similarity)		
ERF-1	Solyc02g077820.1.1	Solyc03g093610.1.1	Solyc02g077820.1.1	Ethylene-responsive transcription factor 1; Involved in the regulation of gene expression during fruit ripening, by stress factors and by components of stress signal transduction pathways. Transcription factor that binds to the GCC-box pathogenesis-related promoter element. Probably acts as a transcriptional activator and may be involved in disease resistance pathways (By similarity)	annotation not available	0.777
ERF-1	Solyc05g050280.2.1	Solyc03g093610.1.1	Solyc05g050280.2.1	Ethylene-responsive transcription factor 1; Involved in the regulation of gene expression during fruit ripening, by	annotation not available	0.697

				stress factors and by components of stress signal transduction pathways. Transcription factor that binds to the GCC-box pathogenesis-related promoter element. Probably acts as a transcriptional activator and may be involved in disease resistance pathways (By similarity)		
ERF-1	Solyc05g050290.1.1	Solyc03g093610.1.1	Solyc05g050290.1.1	Ethylene-responsive transcription factor 1; Involved in the regulation of gene expression during fruit ripening, by stress factors and by components of stress signal transduction pathways. Transcription factor that binds to the GCC-box pathogenesis-related promoter element. Probably acts as a transcriptional activator and may be involved in disease resistance pathways (By similarity)	annotation not available	0.697
LOC544041	101248306	Solyc05g052050.1.1	Solyc06g076160.2.1	Uncharacterized protein; Pti4	annotation not available	0.583

LOC544041	NAC1	Solyc05g052050.1.1	Solyc04g009440.2.1	Uncharacterized protein; Pti4	NAC domain protein; NAC domain protein 1	0.603
LOC544041	Solyc02g077820.1.1	Solyc05g052050.1.1	Solyc02g077820.1.1	Uncharacterized protein; Pti4	annotation not available	0.551
NAC1	101248306	Solyc04g009440.2.1	Solyc06g076160.2.1	NAC domain protein; NAC domain protein 1	annotation not available	0.776
NAC1	101256610	Solyc04g009440.2.1	Solyc08g007230.1.1	NAC domain protein; NAC domain protein 1	annotation not available	0.776
NAC1	ERF-1	Solyc04g009440.2.1	Solyc03g093610.1.1	NAC domain protein; NAC domain protein 1	Ethylene-responsive transcription factor 1; Involved in the regulation of gene expression during fruit ripening, by stress factors and by components of stress signal transduction pathways. Transcription factor that binds to the GCC-box pathogenesis-related promoter element. Probably acts as a transcriptional activator and may be involved in disease resistance pathways (By similarity)	0.777

Supporting Information Table S2 Primers used for quantification of mRNA levels by qRT-PCR

**Gene bank (Accession number)**	**Gene**	**Forward (5'- 3')**	**Reverse (5'- 3')**
NM001247553.3	NAC1	CAAGTTTGATCCATGGCAGTTG	CCGTGAACCGTTCGGATATT
KC447280.1	NAC1.1	GACACCAGCAAACGACTACT	CTGTGGTCTTCGTCCCATTT
JZ714624	Heat shock protein	TTTTCAATATGGGTGCTGGTT	CTGCCAAGGCTTTTATCTGG
JZ714625	Zinc finger protein	CCACCGCTACTCCTTTCACT	GCATGAAGATTGATCCATACGA
GU591574.1	NAC2	ATCCGACAGACGACGAATTG	AAAGTCGCCGCCGATTAG
JZ714627	Late embryogenesis abundant protein	ACGAGTGGAGGAGTGGTGTC	GCAACTGCCATGGATCAAAC
AJ222579	Elongation factor 1 alpha	GACAACATGATTGAGAGGTCCACC	GGCTCCTTCTCAATCTCCTTACC
NM001330119.1	Actin	GTGTCTGGATTGGAGGATCAATC	GGCCACGCTCATCATATTCA
